# London as a Post-Graduation Medical School

**Published:** 1918-07-20

**Authors:** 


### LONDON AS A POST-GRADUATION MEDICAL SCHOOL.

In medical circles a rumour prevails to the effect that an influential movement is being organised with a view to develop in London an adequate scheme of clinical and laboratory teaching for medical practitioners. The proposal is a timely one. Not only is the need for teaching of this order being more and more widely recognised, but as a direct consequence of the war the field to a very large extent is left open. The capital cities of the two Central Empires for many years prior to the war offered special opportunities for post-graduation work to medical practitioners, and these were widely utilised by English-speaking medical men, while London allowed itself to appear as a quite ineffective competitor. The war will certainly alter this state of matters so far as Berlin and Vienna are concerned. It is simply inconceivable that Britons and Americans can for many a day care to visit these cities, whether for purposes of study or otherwise. Thus there will exist a great opportunity, and it remains to be seen whether the medical world of London has the foresight and capacity to grasp it. The clinical material available here cannot be bettered; here are physicians and surgeons of high authority and repute, while the attractiveness of tradition and the bond of language carry an appeal which modern history is emphasising. Manifestly what is wanting is an organised scheme based upon a recognition of the nature of the demand and carried through under the sense of a high and deliberate purpose. The question is—Will medical London prove equal to the occasion? Time is an important element in the decision, and union and the suppression of petty jealousies necessary conditions. The hour is here and now, and neglect may well prove fatal. Both patriotism and professional responsibility may be used as motives for a worthy effort. Personally, we have no doubt of success if only the enterprise is inspired by large views and directed by courageous and liberal minds.

It is well frankly to state that there is no hope except in a bold and inclusive and commanding programme. Earlier schemes have come to naught for many reasons, and one of these is the absence of a hospital having a metropolitan status and devoted purely to post-graduation work. The medical student and the medical practitioner cannot be combined in one and the same scheme of instruction, as all experienced teachers, we doubt not, will readily allow. Nor is it probable that any substantial attraction can be developed in a proposal which would arrange classes sometimes at one school and sometimes at another. Such an arrangement lacks definiteness and precision, and is inconsistent with a whole-hearted devotion and with the growth of inspiring and attractive traditions. There must be a local habitation as well as a name, and this in practice means that one of the existing hospitals must become the post-graduation clinical school. A second practical necessity is that all the capable teaching resources of London must be affiliated to this school. No single hospital staff can provide what is required if the programme is to exercise a world-wide attraction. Not only a hospital but a college is necessary, and within this must be welcomed everyone who has something to teach and who is competent to teach it. We hear of "in and out" proposals at various medical schools, and of brief and intensive courses of instruction. To us such proposals hold out no hope of anything beyond a local and parochial success. What is wanted is a scheme which will insure a comprehensive and enduring appeal, and this means a scheme equal to the opportunities which London may justly boast. The minimum must be nothing less than a hospital entirely devoted to the work, and a platform of teaching marked by efficiency and capacity wherever these qualities are to be found.

